# Novel Hydrogel Scaffolds Based on Alginate, Gelatin, 2-Hydroxyethyl Methacrylate, and Hydroxyapatite

**DOI:** 10.3390/polym13060932

**Published:** 2021-03-18

**Authors:** Simonida Lj. Tomić, Jasmina Nikodinović-Runić, Marija Vukomanović, Marija M. Babić, Jovana S. Vuković

**Affiliations:** 1 Faculty of Technology and Metallurgy, University of Belgrade, Karnegijeva 4, 11000 Belgrade, Serbia; mbabic@tmf.bg.ac.rs (M.M.B.); jjovasevic@tmf.bg.ac.rs (J.S.V.); 2 Institute of Molecular Genetics and Genetic Engineering, University of Belgrade, Vojvode Stepe 444a, 11000 Belgrade, Serbia; jasmina.nikodinovic@imgge.bg.ac.rs or; 3 Advanced Materials Department, Jožef Stefan Institute, Jamova Cesta 39, 1000 Ljubljana, Slovenia; marija.vukomanovic@ijs.si

**Keywords:** alginate/gelatin/2-hydroxyethyl methacrylate/hydroxyapatite, hydrogel scaffolding biomaterial, degradable scaffolds, biocompatibility, tissue regeneration engineering

## Abstract

Hydrogel scaffolding biomaterials are one of the most attractive polymeric biomaterials for regenerative engineering and can be engineered into tissue mimetic scaffolds to support cell growth due to their similarity to the native extracellular matrix. The novel, versatile hydrogel scaffolds based on alginate, gelatin, 2-hydroxyethyl methacrylate, and inorganic agent hydroxyapatite were prepared by modified cryogelation. The chemical composition, morphology, porosity, mechanical properties, effects on cell viability, in vitro degradation, in vitro and in vivo biocompatibility were tested to correlate the material’s composition with the corresponding properties. Scaffolds showed an interconnected porous microstructure, satisfactory mechanical strength, favorable hydrophilicity, degradation, and suitable in vitro and in vivo biocompatible behavior. Materials showed good biocompatibility with healthy human fibroblast in cell culture, as well as in vivo with zebrafish assay, suggesting newly synthesized hydrogel scaffolds as a potential new generation of hydrogel scaffolding biomaterials with tunable properties for versatile biomedical applications and tissue regeneration.

## 1. Introduction

Regenerative engineering aims to repair more complex tissues and biological systems by integrating materials engineering, cell science, and developmental biology [1,2,3]. A major challenge in regenerative engineering is the design and fabrication of a suitable scaffold, which can mimic the native extracellular matrix (ECM) to regenerate functional tissues. In this respect, hydrogels are one of the most promising biomaterials based on their high water content, biocompatibility, and easy tunability for recreating the properties of the ECM [4]. Hydrogels are highly attractive polymeric biomaterials for regenerative engineering, since they can be engineered into tissue mimetic scaffolds to support cell growth due to their similarity to the native extracellular matrix. Advanced technologies have increased the ability to control properties and functionalities of hydrogel scaffolding materials by facilitating biomimetic fabrication of more sophisticated compositions and architectures, thus extending understanding of cell–matrix interactions at the nanoscale. Numerous types of hydrogels with optimized physical and chemical properties have been created for regenerative engineering to repair different tissues [5,6]. Various biophysical cues—stiffness, porosity, and degradation—can be incorporated into hydrogel scaffolds in a spatiotemporally controlled manner to systematically regulate the behavior of cells, including their migration, proliferation, and differentiation [7,8]. Many advanced chemical strategies, and the incorporation of functional materials, have also been proposed to improve the biocompatibility and functionality of hydrogels [9]. Recent research has shown that various combinations of alginate, gelatin, and 2-hydroxyethyl methacrylate, as well as with the addition of apatite can be used for the production of hydrogel scaffolding biomaterials with significant potential in tissue regeneration [10,11,12,13,14].

Alginate is a naturally occurring anionic and hydrophilic polysaccharide. It is one of the most abundant biosynthesized materials [15,16,17,18], derived primarily from brown seaweed and bacteria. Alginate contains blocks of (1–4)-linked β-D-mannuronic acid (M) and α-L-guluronic acid (G) monomers. Typically, the blocks are composed of three different forms of polymer segments: consecutive G residues, consecutive M residues, and alternating MG residues. Alginate is of particular interest for a broad range of applications as a biomaterial and especially as the supporting matrix or delivery system for tissue repair and regeneration. Due to its outstanding properties in terms of biocompatibility, biodegradability, non-antigenicity, and chelating ability, alginate has been widely used in a variety of biomedical applications including tissue engineering, drug delivery, and in some formulations preventing gastric reflux [19,20].

Scaffolds are used as a smart “house” that protects and allows cells to multiply indoors and regenerate tissues. As such, scaffolding materials allow the protection of biologically active agents or cells from the biological environment. Depending on conditions, biomaterials are subjected to different pH environments, which affect the degradation properties, mechanical properties, and swelling behavior of biomaterials. As such, alginate plays an important role in the long-term stability and performance of alginate-based biomaterials in vitro. As a Food and Drug Administration (FDA, Silver Spring, MD, USA) approved polymer, alginate has become one of the most important biomaterials for diverse applications in regeneration medicine and nutrition supplements [21,22,23,24,25]. Since alginate lacks an informational structure for positive cell biological response, the modification and functionalization of synthetically derived alginate hydrogels are usually required.

Gelatin is a molecular derivative of collagen obtained via the irreversible denaturation of collagen proteins. Gelatin shares a very close molecular structure and function with collagen and thus is often used in cell and tissue culture to replace collagen for biomaterial purposes. Recent technological advancements have resulted in great strides toward the generation of functional gelatin-based materials for medical purposes. Gelatin polymer is a well-known biodegradable and biocompatible material that consists of 85–92% of proteins, mineral salts, and water [26,27]. Gelatin contains linear tripeptide Arginine (Arg), Glycine (Gly), and Aspartate (Asp) recognition sequences that bind to several integrin proteins and thus aid in cell attachment, migration, and survival [28]. It is a molecular derivative of Type I collagen and has a wide range of food, cosmetic, biomedical, and pharmaceutical applications [29,30]. It is generally produced by the irreversible hydrolyzation of the triple helical structure of collagen via processes such as heat and enzymatic denaturation, producing random coiled domains. Gelatin has less organization but has a very similar molecular composition to collagen [31]. As a result of this, gelatin has the capacity to replace and perform similar biomaterial functions as collagen for cellular development in vitro. Gelatin is readily available and can be extracted from several sources such as cattle bones, fishes, pig skins, and some insects. Several studies on the biocompatibility of gelatin derived from various sources showed that gelatin, in general, does not induce toxicity, antigenicity, and other adverse effects in human cells. Thus, the use of gelatin has gained popularity over pure and intact ECM proteins for the following reasons: it is more readily available and much cheaper than ECM proteins, highly soluble compared to other ECM proteins, and thus is easier to use for biomedical purposes. Only a few sections of the full ECM protein sequence are important for cell attachment and eliciting the cellular response; thus, the use of intact proteins may not be necessary. Gelatin possesses a highly similar structure to collagen and contains important binding moieties for cell attachment; different gelatin sources are biocompatible, biodegradable, and do not induce antigenicity and toxicity in cells. However, for biomaterial purposes, gelatin also possesses some disadvantages. The main drawback of using gelatin is that gelatin-based materials have poor mechanical properties, lack thermal stability, and have a relatively shorter degradation rate. When used in studies that require a longer time such as controlled drug release, cell differentiation, and wound healing, gelatin-based materials may not last. Moreover, compared to collagen, gelatin is highly susceptible to several proteases and thus may lead to its faster degradation [32]. However, these disadvantages can be easily overcome by modifying gelatin and making gelatin composites to increase the material’s mechanical stability, biocompatibility, and bioactivity. The advancement of manufacturing technology and our knowledge of material chemistry have made these drawbacks less important compared to the limitless benefit of using gelatin for biomedical purposes.

2-Hydroxyethyl methacrylate (HEMA) is a famous monomer due to its versatility for the synthesis of polymeric biomaterials: hydrogels and hydrogel scaffolds. HEMA-based polymeric materials show favorable biocompatibility and tunable hydrophilicity. Therefore, they have multiple applications in pharmaceutical and biomedical fields such as coatings, intraocular lenses, scaffolds, and devices for controlled drug release. HEMA-based hydrogels are mainly combined with hydrophilic polymer components to tune biocompatibility, swelling, and mechanical properties [33,34,35].

Hydroxyapatite (HAp) is an inorganic agent and due to its favorable biocompatibility and biological functionality is used in tissue regeneration [36,37,38]. However, the main limitation for the use of HAp ceramics was their inherent brittleness and difficulty for processing [39]. To combine mutually beneficial properties, polymer/inorganic scaffolds have been developed for tissue engineering either by direct mixing or by a biomimetic approach [40]. Compared to plain polymeric scaffolds in which neotissue matrix was formed only in the surface layer [35], composite scaffolds containing apatite supported cells growth and neotissue formation throughout the scaffold including in the very center of the scaffold [41].

In this work, we set a hydrogel scaffolding biomaterials platform of porous scaffolds based on alginate, gelatin, 2-hydroxyethyl methacrylate, with and without inorganic agent hydroxyapatite, which was fabricated by cryogelation and characterized to show that the proposed technique of synthesis provides favorable mechanical, morphological, and degradation properties, as well as in vitro and in vivo suitable biocompatible behavior, which is crucial to provide a desirable environment for the viability and survival of cells. The effect of the composition of hydrogel scaffolds on the aforementioned properties was investigated as well.

## 2. Materials and Methods

### 2.1. Materials

2-Hydroxyethyl methacrylate (HEMA, Sigma-Aldrich, St. Louis, MO, USA), ethyleneglycol dimethacrylate (EGDMA) (Aldrich), potassium persulfate (PPS) (Fluka), and *N*, *N*, *N*′, *N*′-tetramethylene diamine (TEMED) (Sigma-Aldrich) were used as a monomeric component, crosslinking agent, initiator, and activator, respectively. Natural origin polymeric components, gelatin (type B from bovine skin) (Sigma-Aldrich), and sodium alginate (Sigma-Aldrich), as well as crosslinker 1-ethyl-3-(3-dimethyl aminopropyl) carbodiimide hydrochloride (EDC) (Sigma-Aldrich) were used for hydrogel scaffolds syntheses. The deionized water was used as a solvent.

Materials used for the hydroxyapatite synthesis and doping were calcium nitrate pentahydrate (Ca(NO_3_)_2_ × 5H_2_O) (Sigma-Aldrich), magnesium nitrate hexahydrate (Mg(NO_3_)_2_ × 6H_2_O) (Sigma-Aldrich), strontium nitrate (Sr(NO_3_)_2_) (Sigma-Aldrich), gallium nitrate hydrate (Ga(NO_3_)_3_ × H_2_O) (Sigma-Aldrich), zinc nitrate hexahydrate (Zn(NO_3_)_2_ × 6H_2_O) (Sigma-Aldrich), ammonium dihydrogen phosphate (NH_4_H_2_PO_4_) (Sigma-Aldrich), and urea ((NH_2_)_2_CO) (Alfa Aesar). All chemicals and reagents were of analytical grade. All experiments were performed using lab-produced, ultra-distilled water.

### 2.2. Synthesis of Hydrogel Scaffolds

Scaffolding materials based on alginate, gelatin, and 2-hydroxyethyl methacrylate were synthesized via cryogelation, with corresponding agents for gelation. A set of syntheses was performed by varying the content of components. Based on that, the optimal composition of the samples was obtained: gelatin/HEMA = 0.2/0.8 and alginate/gelatin/HEMA = 0.1/0.1/0.8 weight ratio (prepared samples were designated as GH and A50G50H). The components were added simultaneously into a test tube and vigorously stirred at 63 °C; afterwards, the reaction mixture was cooled to −18 °C for 24 h. After synthesis, hydrogel samples were cut into disks and immersed in deionized water for 7 days to remove unreacted components. Then, the hydrogel disks were dried to constant weight. After that, the disks were lyophilized and used for further characterizations.

#### 2.2.1. Synthesis Route of Hydroxyapatite

Multidoped apatite was synthesized using the sonochemical homogeneous precipitation method with thermally degraded urea [42]. Ca- and dopants (Zn-, Sr-, Ga- and Mg-) precursors were pre-mixed in an equal molar ratio (1:1:1:1). After the addition of a P-precursor (2 wt %), the mixture was preheated to 80 °C, which was followed by adding urea (12 wt %). Intensive sonification (with pulsation-to-relaxation periods on/off = 02:01 s, power P = 600 W, frequency f = 20 kHz and amplitude A = 80%) was continued for the next three hours. During that period, the gradual thermal decomposition of urea led to precipitation. Upon finishing sonification, the precipitate was aged in the supernatant for the next 15 h (under ambient conditions) which was followed by centrifugation (10 min at 6000 rpm) and air drying. The synthesis was performed using an ultrasonic processor for high-volume applications (VCX 750).

#### 2.2.2. Incorporation of Hydroxyapatite in Hydrogel Scaffolds

Hydroxyapatite doped with Mg, Sr, Zn, Ga, and Au/Arginin was incorporated (5% of total hydrogel disk weight) during hydrogel synthesis, while the reaction mixture was vigorously stirred to homogenously distribute HAp particles. Hydrogel scaffolds loaded with HAp were designated as GH/HAp and A50G50H/HAp.

### 2.3. Characterization of Hydrogel Scaffolds

#### 2.3.1. Fourier Transform Infrared Spectroscopy (FTIR)

The chemical composition of hydrogel scaffolds was analyzed using FTIR spectra recorded on an FTIR Nicolet 6700 (Thermo-Scientific) diamond crystal spectrometer with attenuated total reflectance (ATR) sampling technique. FTIR spectra were recorded over the wavelength range of 700–4000 cm^−1^.

#### 2.3.2. Scanning Electron Microscopy (SEM)

A scanning electron microscope (JEOL JSM-5800 LV) was used to observe the morphologies of the xerogels. Hydrogel samples were lyophilized, fixed on a titanium carrier, and then gold sputter-coated under vacuum before observation.

#### 2.3.3. Porosity

The porosity of hydrogel scaffolds was calculated based on bulk and true density of the materials:

(1) $$\pi =1-\rho_{hydrogel}/\rho_{bulk}$$

using density determined by the Archimedes method. Glycerol (with a density *ρ*_0_ = 1.2038 g/cm^3^) was used as a wetting medium. The samples were weighted under ambient conditions before (*m_a_*) and after (*m_b_*) immersion into the liquid and calculations were performed using the formula:

(2) $$Porosity=m_{a}/(m_{a}+m_{b})\times \left(\rho_{0}-\rho_{L}\right)+\rho_{L}$$

where *ρ_L_* is the density of air (*ρ_L_* = 0.0012). The final values are the average of three measurements.

#### 2.3.4. Mechanical Properties Testing

Mechanical properties of the hydrogels were tested using a universal testing machine (Galdabini Quasar 50) by the application of a uniaxial compression with a 100-N load cell at room temperature. Obtained Young’s modulus (*E*) was calculated from the linear part of the stress/strain curve, and its final value is the average of three measurements.

#### 2.3.5. Water Contact Angle

The static water contact angle measurement was measured using the sessile drop method by placing a drop (approximately 1 μL) of MilliQ water on the surface of the hydrogel. The measurements were performed using a Contact angle meter Theta Lite- Biolin Scientific (with measuring range 0–180 deg. and accuracy +/− 0.1 deg., +/− 0.01 mN/m) equipped with the camera with 640 × 480 resolution and maximum measuring speed 60 fps. All measurements were repeated at least four times for each hydrogel.

#### 2.3.6. In Vitro Degradation Study

In vitro degradation studies were conducted by immersing hydrogels in phosphate buffer of pH 7.40, at 37 °C, for 3 months. After incubation, samples were removed from the degradation media, washed with deionized water several times to remove the excess salts, oven-dried to constant mass, and measured. Degradation was presented as the weight loss of the samples, which was calculated by the following equation [43]:

(3) $$\mathrm{Weight}\;\mathrm{loss}\;=\;\frac{\left(m_{0}-m_{t}\right)}{m_{0}}\times 100\%$$

where *m*_0_ (*m*_0_) is the initial weight of the dry hydrogel, and *m_t_* is the weight of the dried hydrogel at the time of measuring.

#### 2.3.7. Biocompatibility Studies

##### In Vitro Assay

Cytotoxicity (antiproliferative activity) was measured for MRC5 cells (human lung fibroblast, obtained from ATCC) using a standard MTT assay (MTT assay is used to measure cellular metabolic activity as an indicator of cell viability, proliferation and cytotoxicity–this colorimetric assay is based on the reduction of a yellow tetrazolium salt (3-(4,5-dimethylthiazol-2-yl)-2,5-diphenyltetrazolium bromide or MTT) and methods suitable for materials testing [12,44,45,46,47,48,49,50].

##### In Vivo Toxicity Assessment Using Zebrafish

Ethical issues related to animal experiments are handled according to the signed statement of the Animal Ethics Committee of the Institute of Molecular Genetics and Genetic Engineering (IMGGE) in accordance to Directive 2010/63 of the European Parliament on the protection of animals used for scientific purposes and the Law on Environmental Protection of the Republic of Serbia (the Animal Welfare Act; Official Gazette of the Republic of Serbia No. 41/2009).

Evaluation of toxicity of the tested biomaterial in the zebrafish model was carried out according to universal rules of the OECD Guidelines for the Testing of Chemicals [51]. All experiments involving zebrafish were performed in compliance with the European directive 2010/63/EU and the ethical guidelines of the Guide for Care and Use of Laboratory Animals of the Institute of Molecular Genetics and Genetic Engineering, University of Belgrade.

## 3. Results and Discussion

**Scheme 1:**
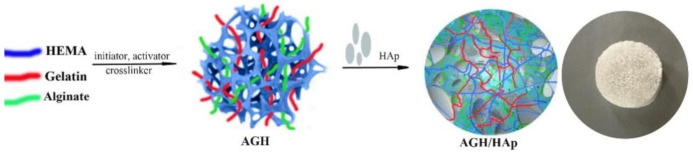
Schematic representation of the formation of the polymeric hydrogel scaffolds composed of alginate, gelatin, 2-hydroxyethyl methacrylate (HEMA) and hydroxyapatite (Hap).

Novel, porous, and degradable hydrogel scaffolds were successfully synthesized by combining natural polymers, alginate and gelatin, and synthetic monomeric component HEMA (AGH) loaded with an inorganic agent, HAp (AGH/HAp), using cryogelation. The synthesis route of the hydrogel scaffolds is shown in Scheme 1.

### 3.1. Structural Characteristics

**Figure 1:**
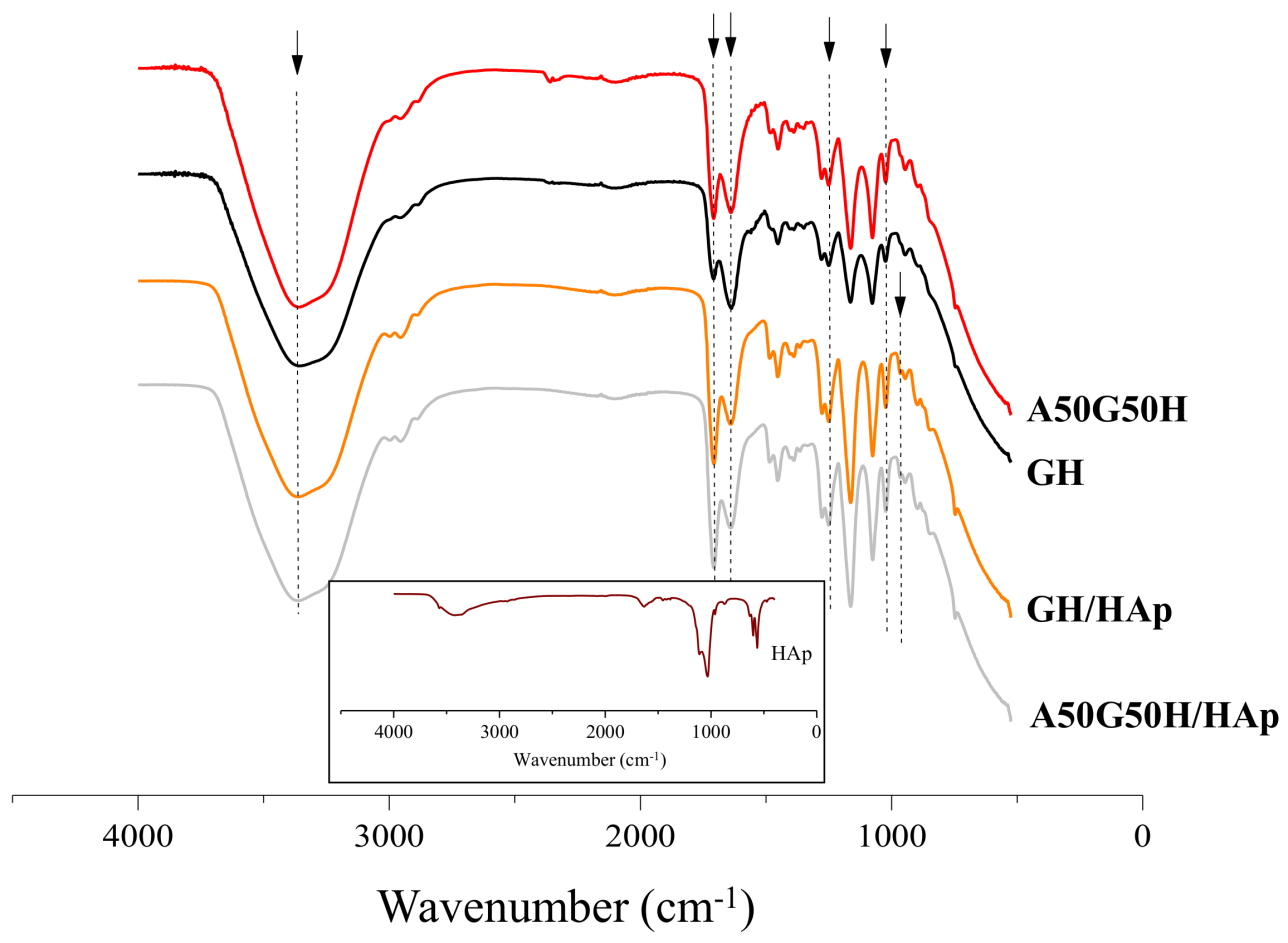
Fourier transform infrared spectroscopy (FTIR) spectra of hydrogel scaffolds (insert is FTIR spectrum of HAp).

Fourier transform infrared spectroscopy (FTIR) provided an insight into structural characteristics of scaffolds based on alginate, gelatin, HEMA, and HAp. FTIR spectra, presented in Figure 1, were recorded to identify the characteristic bands of the functional groups that originated from hydrogel components (gelatin, alginate, HEMA, and HAp). The peaks related to HEMA around 3360, 2940, 1640, and 1710 cm^−1^ were assigned to asymmetric and symmetric stretching vibrations (terminal hydroxyl group), methylene stretching, terminal vinyl, and a carbonyl group. Furthermore, the peaks at 1640 cm^−1^ and 1240 cm^−1^ were assigned to amide I and amide III, while the characteristic peak of amide II bands of gelatin at 1540 cm^−1^ [52] failed to be detected. Additionally, the peak at 1640 cm^−1^ can be assigned to asymmetric stretching of -COO− of alginate [53].

The appearance of a peak at 965 cm^−1^, related to the asymmetric stretching mode of P-O of the PO_4_^3−^ group, and change of the intensity of peaks at 3360, 1710, 1640, and 1240 cm^−1^ confirmed the presence of Hap incorporated in scaffolds [54,55]. Apatite crystalized as a single phase with hexagonal structure (PDF 01-089-6438) and its FTIR spectrum contains all characteristic bands, including vibrations of PO_4_^3−^ groups at 472, 583, 601, 961, 1032, and 1108 cm^−1^ and OH^-^ vibrations at 3573 and 632 cm^−1^ (an insert in Figure 1) [56].

The identification of the peak characteristics for each component used for hydrogel scaffolds preparation confirmed successful syntheses, as well as the incorporation of HAp in the complex polymeric matrices based on alginate, gelatin, and HEMA.

### 3.2. Morphology of Hydrogel Scaffolds

Polymeric porous biomaterials should be able to provide architecture essential for cells attachment, differentiation, and proliferation, and ultimately the formation of novel tissue. Thus, the constructions of well-defined interior and exterior microenvironment of the biomaterial must be carefully and precisely designed [57]. The porosity is the governing factor in choosing a suitable biomaterial for tissue engineering applications. High porosity with an open interconnected geometry allows a large surface area relative to the scaffold’s volume, which is directly related to the function of the biomaterial. To control and tailor the porosity means to have an insight into the homogeneity of such micro-architecture, the size and shape of the pores, as well as their orientation and interconnectivity [58].

**Figure 2:**
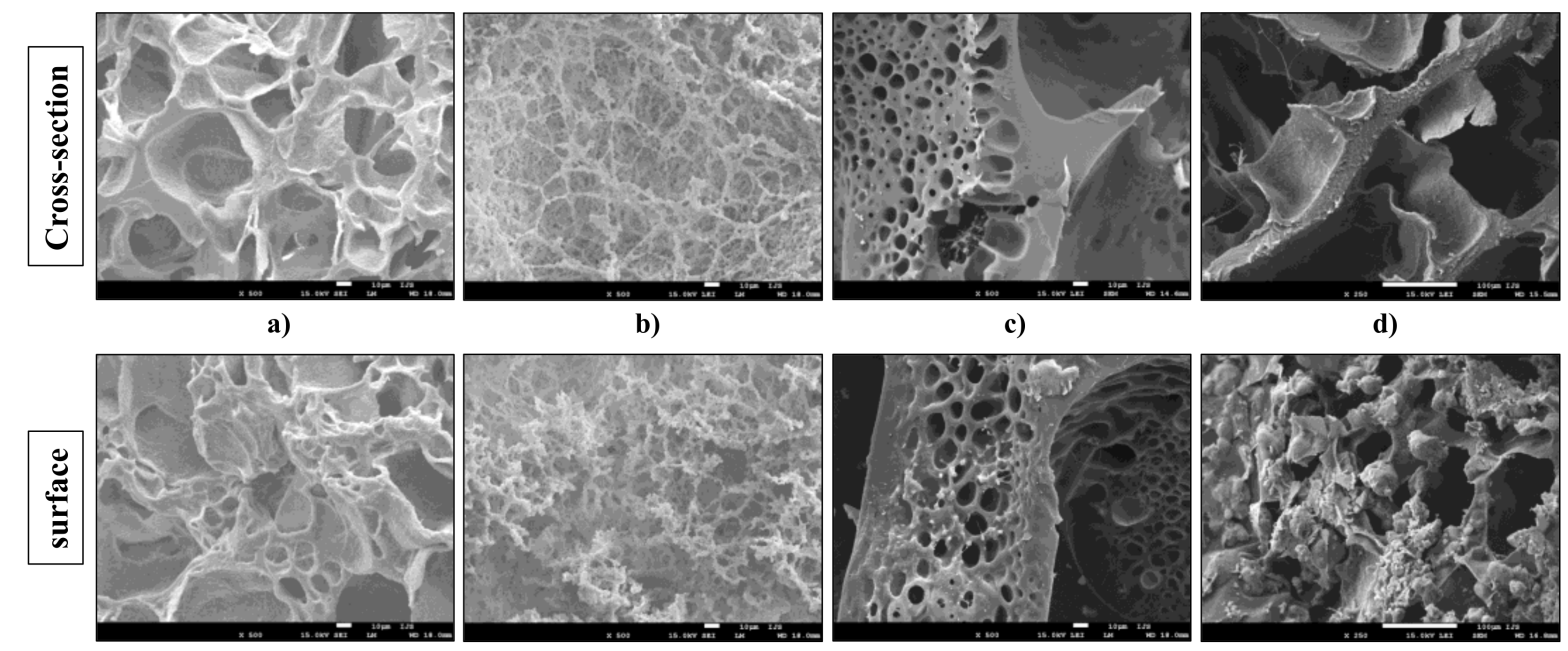
SEM micrographs of cross-section and surface of (**a**) GH, (**b**) A50G50H, (**c**) GH/HAp, and (**d**) A50G50H/HAp samples (smaller bar 10 μm, larger bar 100 μm).

The morphology of hydrogel scaffolds based on alginate, gelatin, HEMA, and HAp was investigated using scanning electron microscopy, whereby both the cross-sections and surfaces of the samples were thoroughly examined. The obtained SEM micrographs of the scaffolds are presented in Figure 2.

According to the obtained micrographs, hydrogel scaffolds possess a porous structure with interconnected pores. The pores are different in sizes, shapes, and orientations. In addition, there were no indicators for any phase separation, which confirmed the good compatibility between the components used in scaffold syntheses. The inner morphology of the GH scaffold is reflected in its homogeneous porous architecture with interconnected open pores with circular and irregular geometries. The polymeric construction of scaffolds based on gelatin and HEMA can provide the efficient nutrient distribution crucial for the vascularization, proliferation, and differentiation of the cells. Likewise, the micrographs of the surface of GH scaffold revealed the different size of pores and their interconnections, suggesting that the detected surface porosity can enhance the interactions between these biomaterials, which aim for tissue regeneration, and cells, implying their faster attachment. The presence of alginate in the A50G50H scaffold caused morphological changes. The structure became denser, the shape of pores became more irregular, and alginate as an interpenetrating component of scaffold was noticeable in the form of a coating on the pore walls. The same phenomenon, almost similar to coral architecture, was revealed on the surface of the A50G50H scaffold.

The micrographs of the cross-section and surface of the GH/HAp scaffold revealed the presence of HAp incorporated in the polymeric matrix. The visible particles confirmed the successful loading of the inorganic bioactive agent. Furthermore, the thicker structure of the GH/HAp scaffold possesses smaller pores with a more regular, circular shape, indicating the reinforcement caused by the presence of HAp. On the other hand, the morphology of A50G50H/HAp scaffold became laminar and more irregular. The micrograph of the surface of A50G50H/HAp scaffold revealed visible aggregations, which could be the consequence of HAp retained on the surface of the sample and its interactions with alginate. These foundings could result in the weaker structure of the A50G50H/HAp scaffold, affecting the overall mechanical strength, regardless of the incorporation of HAp.

### 3.3. Porosity of Hydrogel Scaffolds

The porosity of the biomaterials aimed for tissue regeneration application plays an important role in cell growth and tissue formation. The optimal porosity of the biomaterial is often mandatory for homogeneous cell distribution and the diffusion of nutrients and oxygen [59].

**Figure 3:**
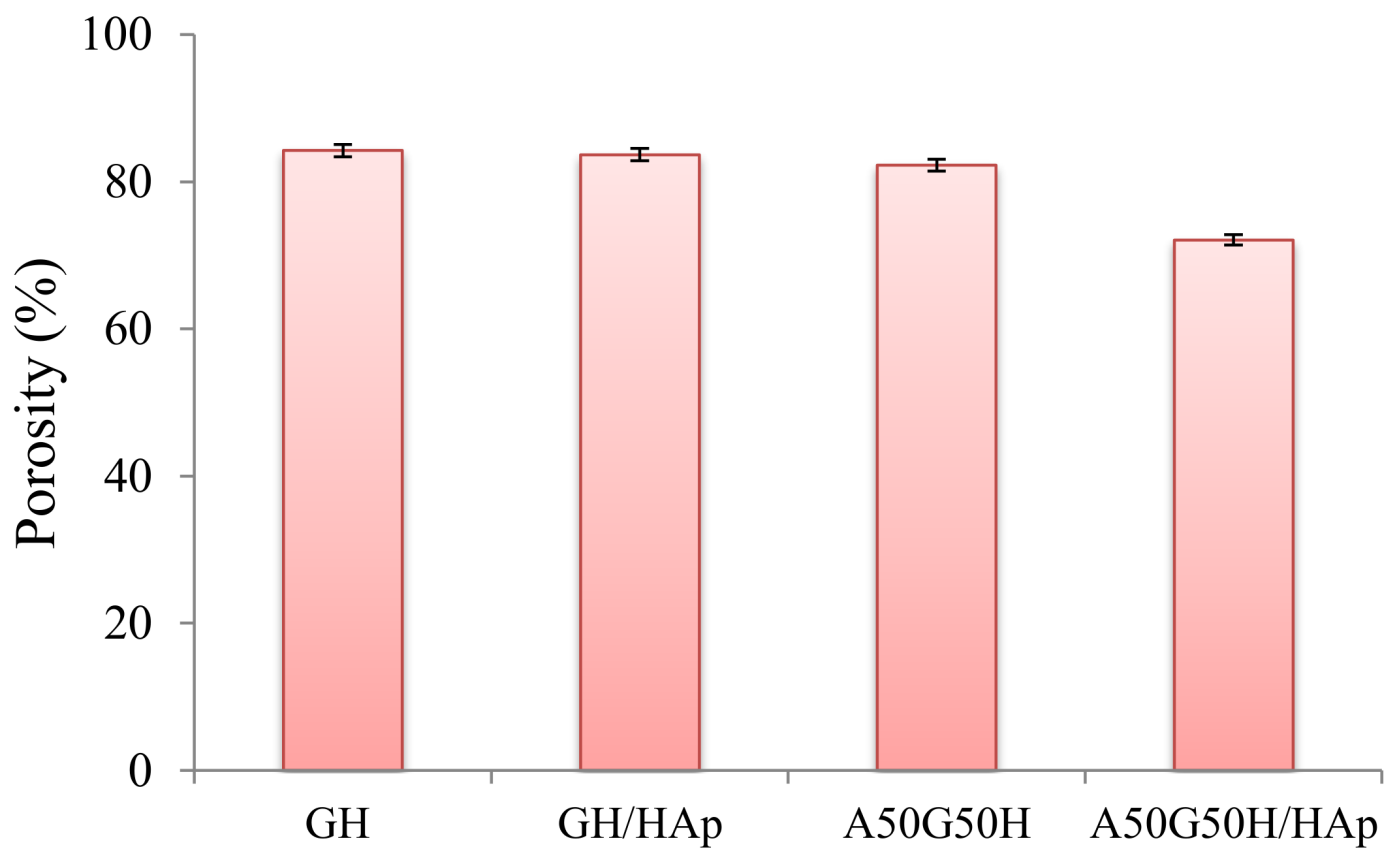
Porosity of hydrogel scaffolds.

The values of porosity of the hydrogel scaffolds, calculated by Equations (1) and (2), are displayed in Figure 3. According to the obtained results, the porosity of the scaffolds varied in the range of 72.10–84.25%, depending on the chemical composition of hydrogel scaffolds. The highest porosity was shown in the GH sample. As expected, the addition of alginate in the polymeric matrix, as an interpenetrating component of the scaffold, slightly lowered the porosity. Alginate penetrated through the pores of the primary matrix, which was formed of HEMA and gelatin, and filled some of them, causing a decrease of overall porosity.

Likewise, the same phenomenon can be observed for samples containing HAp loaded in a polymeric network. The loading of hydroxyapatite caused the reduction of porosity of the hydrogel scaffolds, which is in accordance with earlier research [14,60,61,62]. The porosity decrease was more pronounced in the case of A50G50H/HAp, confirming the interaction between functional groups of alginate and HAp.

Still, according to the results obtained from the porosity study, the prepared scaffolds can be classified as highly porous biomaterials which can allow the cells to infiltrate and attach to the scaffolding biomaterial, to provide a high surface area-to-volume ratio for polymer–cell interactions, and to obtain minimal diffusion constraints during cell culture. The significance of higher porosity and interconnectivity between the pores is a crucial factor for scaffolds, in such a manner that some researchers think that the porosity should reach even 90% [63,64,65,66].

### 3.4. Mechanical Properties of Hydrogel Scaffolds

The fundamental goal of regenerative engineering is to design ECM-like scaffolding materials with desired optimal mechanical strength to provide mechanical and shape stability and biomechanical stimulation of encapsulated cells to generate an effective engineered tissue. The scaffolding biomaterials made from natural polymers are usually soft, weak, and fragile; thus, the proper reinforcement is strongly recommended. Hydroxyapatite, as an inorganic agent, is expected to provide significant improvement of mechanical strength, as well as the enhancement of bioactivity and biocompatibility, due to its inorganic, non-toxic, and non-immunogenic nature [67].

**Figure 4:**
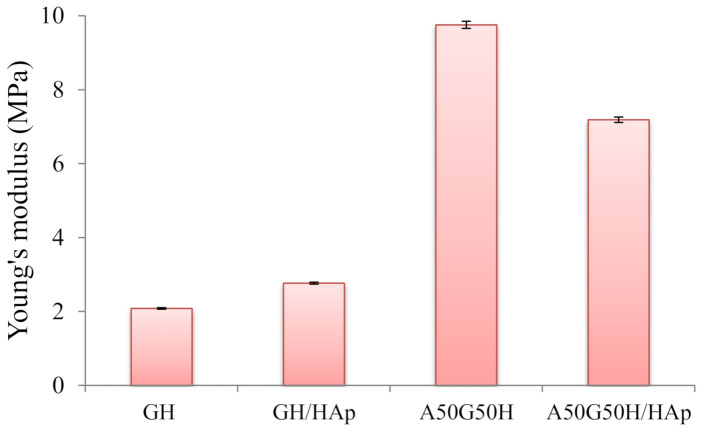
Young’s modulus of hydrogel scaffolds.

To determine the interrelationship between mechanical strength and the composition of the hydrogels, the uniaxial compression experiment was used for all samples. The values of Young’s modulus for hydrogel scaffolds were calculated (Figure 4). As expected, there were variations in the calculated values depending on the scaffold’s composition and the presence of incorporated HAp. Firstly, the addition of alginate resulted in a more compact polymeric structure and increased Young’s modulus, so A50G50H exhibited higher mechanical strength (9.75 MPa) in comparison with the GH sample (2.08 MPa). The obtained results of mechanical testing indicate that introducing alginate can greatly improve the mechanical properties of the synthesized scaffolds, further implicating that the fine tuning of the chemical composition of scaffolds can provide desired mechanical properties for the specific biomedical application.

The incorporation of HApin scaffolds resulted in the reinforcement of GH sample, where the value of the Young’s modulus was raised from 2.08 up to 2.76 MPa. The enhancement of mechanical properties of the scaffold loaded with HAp suggests that there are some interactions between the polymeric matrix and HAp. It is expected that polymeric chains will interact with HAp and attach to their surfaces [68]. The increase of mechanical strength of scaffolds demonstrates good interfacial compatibility between the polymeric matrix and HAp [69].

On the other hand, the loading of HAp agent in A50G50H affected the mechanical properties of the scaffold in the opposite direction. The observed decrease of Young’s modulus of A50G50H/HAp (7.19 MPa) can be elucidated as a consequence of the agglomeration of HAp particles due to their high surface energy, causing the deterioration of mechanical properties [70], which is detected in the SEM micrograph of the A50G50H/HAp sample. This phenomenon is noticed in hybrid materials based on alginate, where the aggregation of HAp particles in the alginate matrix can occur due to low interface compatibility [71]. In addition, at lower concentrations, HAp is distributed more homogenously, and due to the calcium content of HAp, intermolecular linkages are formed between HAp and alginate. However, higher weight fractions of HAp can induce large agglomerations, impairing the material dynamic response and decreasing the mechanical strength [72].

### 3.5. Hydrophilicity of Hydrogel Scaffolds

**Figure 5:**
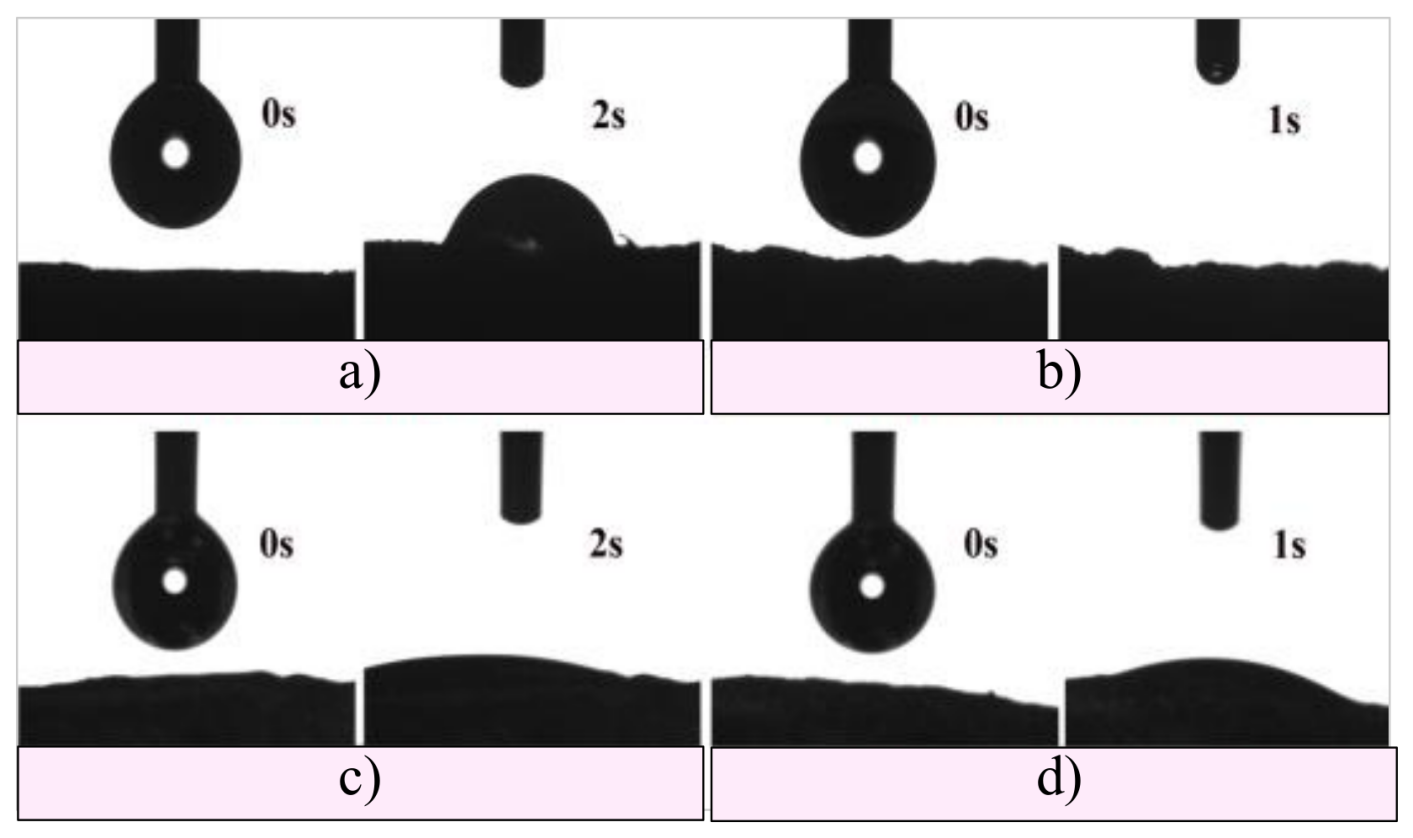
Hydrophilicity of (**a**) GH, (**b**) A50G50H, (**c**) GH/HAp, and (**d**) A50G50H/HAp hydrogel scaffolds.

In the last decades, the development of biomaterials has been focusing on modifications of their surface to promote a greater understanding and control of the performance of material for improving biocompatibility. The surface hydrophilicity is well known as a key factor to govern the response of cells. On hydrophilic surfaces, cells generally showed good adhesion, spreading, proliferation, and differentiation. The water surface contact angle measurements were used to determine the surface hydrophilicity of the scaffolds, and the obtained results are presented in Figure 5. As can be seen, tested samples (measurements performed at 0, 1, and 2 s) were fully hydrophilic, water completely wetted their surface, and drops immediately disappeared after being put on the surface of the scaffolds. In addition, it was noticed that the water drops had a slightly slower spread on the surface of the GH sample. The obtained results showed a large adhesion between the contact surfaces, suggesting that hydrogel scaffolds are ideal for applications where the hydrophilic surface is required, enabling better cell attachment and enhanced biological response.

### 3.6. Degradation Behavior of Hydrogel Scaffolds

**Figure 6:**
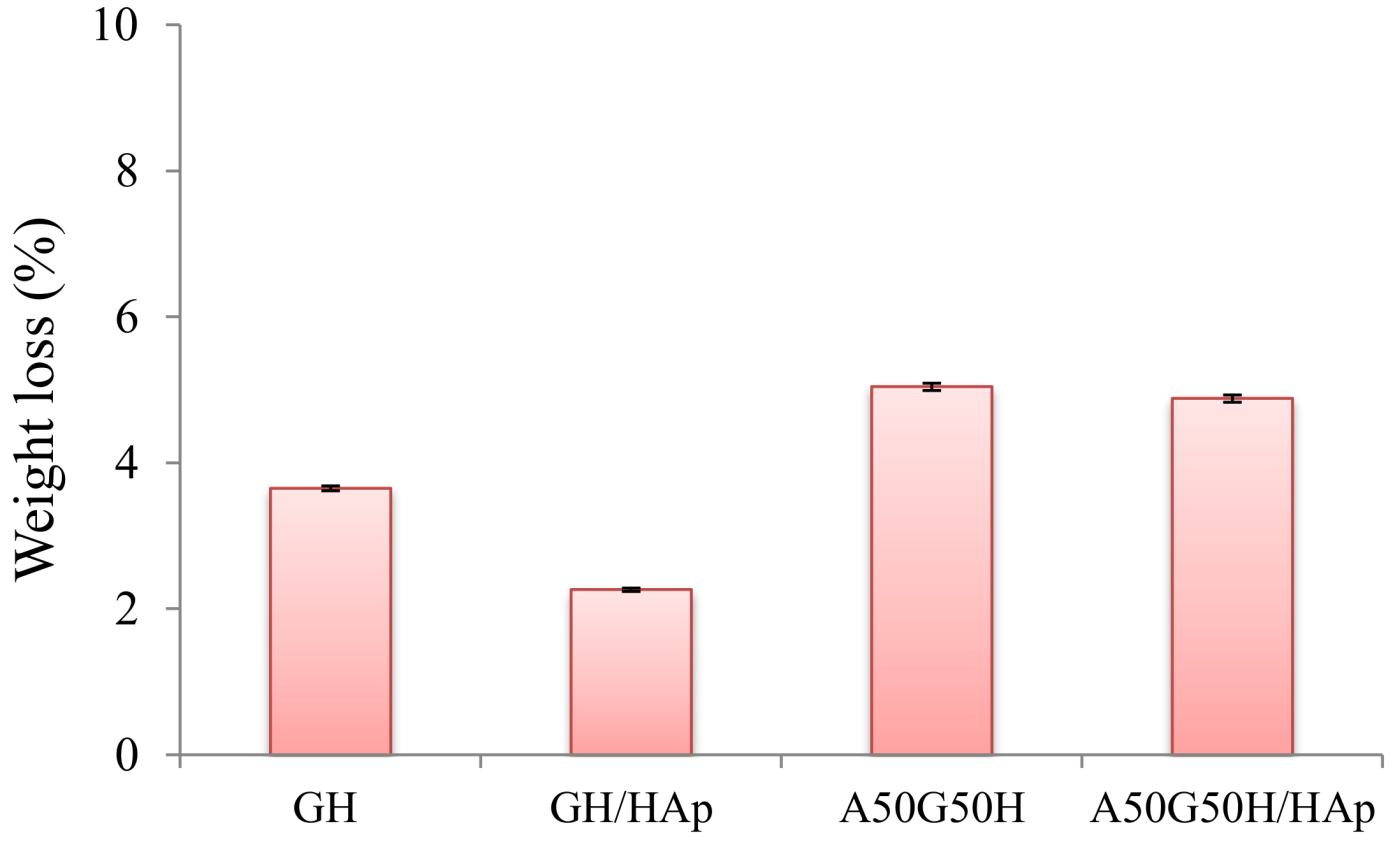
Weight loss during the degradation of hydrogel scaffolds.

The degradation of a biomaterial aimed for tissue regeneration is a requirement for downstream cell differentiation and functional tissue integration, which determines the therapeutic outcome; thus, these materials should be able to degrade within a certain time frame [73]. Most of the degradation processes comprehend the water-induced hydrolysis, where the water, as a biological environment, enters the polymeric matrix, initiates its swelling, and triggers the formation of oligomers and monomers. Therefore, the oligomers and monomers leave the polymeric matrix, causing the decrease of weight of the polymeric biomaterial. In addition, the degradation of biopolymer presents a chain scission process, whereby the polymeric chains are cleaved and the oligomers and monomers are created [74]. An in vitro degradation study conducted for up to 3 months initially showed that scaffolds based on alginate, gelatin, and HEMA maintained their structural integrity without completely losing the original architecture and strength. The results were obtained as values of weight loss (%) of the tested samples during 3 months of the degradation study (Figure 6). According to the values of weight loss (%), the influence of the chemical composition of scaffolds is noticeable. The introduction of alginate influenced the degradation by raising it (5.04%) compared to the GH sample (3.65%). This finding could be described as a consequence of alginate leaving the polymeric matrix, considering the way of its interpenetrating incorporation. Thus, degradation behavior (weight loss) can be easily triggered by varying hydrogel scaffold composition. On the other hand, the incorporation of HAp slowed down the degradation process, so GH/HAp exhibited a weight loss of 2.26%, while A50G50H/HAp lost 4.88% of its total weight. This phenomenon could be explained by the ability of HAp to absorb water molecules and make a “shield”, which will slow down the degradation [75,76]. In addition, the agglomeration of HAp may reduce the specific area; thus, the degradation rate of the scaffolds can be reduced [77].

### 3.7. Biocompatibility of Hydrogel Scaffolds

**Figure 7:**
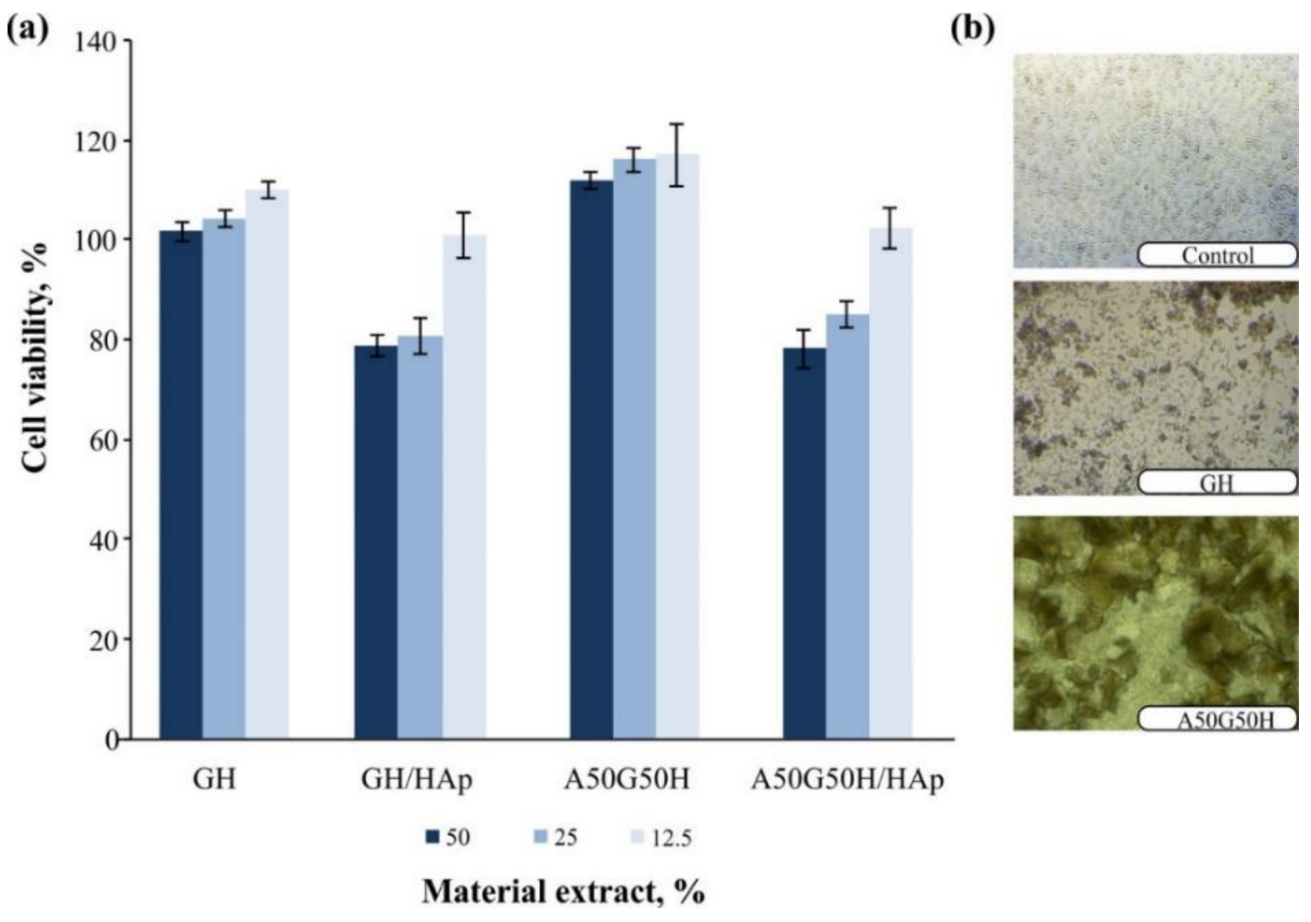
Cell viability upon treatment with extracts of AGH and AGH/HAp hydrogel scaffolds (**a**) and accumulation of cells on its surface (**b**). AGH: alginate, gelatin, and synthetic monomeric component HEMA.

The main focus during the design of the scaffolding polymeric biomaterials to be used in regenerative medicine is the first step related to testing the biocompatibility of the matrix in an in vitro and in vivo system. The biocompatibility of hydrogel scaffolds based on alginate, gelatin, and HEMA, loaded with HAp, was examined in a cytotoxicity test on normal human fibroblasts (MRC5). The results of this assay for samples are shown in Figure 7. The GH, GH/HAp, A50G50H, and A50G50H/HAp samples showed favorable cell viability in contact with cell culture. The loading of HAp into scaffolds has been shown to lead to a slight decrease in cell viability. All materials when supplied directly to the cells were shown to support the accumulation of cells on its surface (Figure 7). The obtained values confirm that AGH and AGH/HAp hydrogel scaffolds show a high level of cytocompatibility, and therefore, they are suitable for applications as biomaterials in tissue regeneration engineering.

To address whether alginate, gelatin, HEMA, and HAp-based materials could be used for biomedical applications, we evaluated their toxicity in vivo using the zebrafish model. The zebrafish model was accepted as a valid alternative to mammalian models (rats mice) for toxicity and biocompatibility evaluation of novel biomaterials, nanomaterials, and drug carriers owing to the high molecular, genetic, physiological, and immunological similarity to humans, and good correlation in response to pharmaceuticals and bioactive compounds [45,78], thus simplifying the path to clinical trials and reducing the failure at later stages of testing [79].

**Figure 8:**
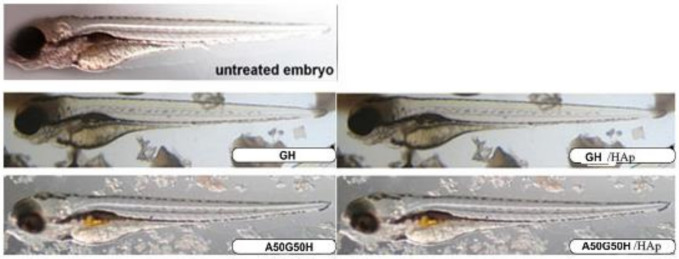
Morphology of zebrafish embryos exposed to the ground materials GH, GH/HAp, A50G50H, and A50G50H/HAp hydrogel scaffolds (200 µg/mL) at 104 hpf.

Given that the organisms at embryonic developmental stages are more sensitive to the chemical insults than as an adults, we exposed zebrafish embryos at the 6 hpf (hours post fertilisation) stage indirectly to the material’s extracts (embryo water in which the ground materials were extracted over 72 h at 37 °C and 180 rpm and applied as diluted suspensions) and to the ground material (200 µg/mL) for 5 days. The obtained data showed that the tested scaffolding materials were not toxic to zebrafish embryos, because there was no lethality, developmental malformations, cardiovascular disorders, or signs of hepatotoxicity (liver necrosis and yolk retention) (Figure 8). It can be said that the synthesized materials are acceptable for biomedical applications. It has been shown following previous research that polymeric biomaterials containing natural polymers can be applied as safe biomaterials for the food packaging and oral delivery to zebrafish larvae or adults [80,81] and more recently as the carriers of drugs to improve their therapeutic outcome (controlled release, reduced toxicity, increase in efficacy) [82,83].

## 4. Conclusions

The novel biocompatible, porous, and degradable hydrogel scaffold platform based on alginate, gelatin, and HEMA, integrated without and with inorganic agent HAp, was successfully synthesized by the simple and effective method, cryogelation. To achieve a balance between the biological and mechanical properties of obtained hydrogel scaffolds to satisfy their final application as promising tissue regeneration engineering scaffolds, the chemical composition of hydrogels was set. The incorporation of alginate and apatite has been shown to affect mechanical properties. Apatite incorporation has a significant effect on porosity as well as on the level of degradation. All hydrogel scaffolds manifested fully hydrophilic surfaces, which make them favorable as scaffolding biomaterials for the adhesion, proliferation, and differentiation of various types of cells. Since PHEMA is biocompatible but not biodegradable, the results obtained from the degradation study for 3 months showed that alginate, gelatin, and HAp improved the degradability of HEMA-based hydrogel scaffolds. In vitro cell culture and an in vivo zebrafish model study confirmed that the hydrogels ensure suitable biocompatibility. All these unique properties indicate that newly designed alginate, gelatin, HEMA, and HAp-based hydrogel scaffolds are promising candidates for regenerative engineering scaffolding biomaterials.
